# A Group of Olfactory Receptor Alleles that Encode Full Length Proteins are Down-Regulated as Olfactory Sensory Neurons Mature

**DOI:** 10.1038/s41598-020-58779-w

**Published:** 2020-02-04

**Authors:** Richard C. Krolewski, Brian Lin, Samuel Stampfer, Adam Packard, James E. Schwob

**Affiliations:** 1 Program in Cell Molecular and Developmental Biology, Graduate School of Biomedical Sciences, Tufts University, Boston, United States; 2 Program in Biochemistry, Graduate School of Biomedical Sciences, Tufts University, Boston, United States; 3 MD-PhD Program, Tufts University School of Medicine and Graduate School of Biomedical Sciences, Tufts University, Boston, United States; 4 Department of Developmental, Molecular and Chemical Biology, Tufts University School of Medicine, Tufts University, Boston, United States

**Keywords:** Adult neurogenesis, Transcription

## Abstract

The family of olfactory receptors (ORs) subserves the sense of smell and includes both functional alleles and pseudogenes, the latter identified by mutations resulting in frame shift or premature truncation. During neuronal differentiation, nonfunctional ORs are expressed initially but then are switched out, and/or the olfactory sensory neurons (OSNs) expressing them die. We carried out a transcriptomic analysis of FACS-isolated cells from *ΔSox2-eGFP*, *Neurog1-eGFP* BAC and *ΔOMP-eGFP* strains of uninjured and olfactory bulbectomized transgenic mice that correspond to distinct stages in the progression from globose basal cell stem cells to fully mature OSNs. We analyzed the expression pattern of 1094 unique receptors across this progression and found that the vast majority were characterized by a typical and expected pattern of expression; i.e., levels of OR mRNA peaking in mature OSNs. However, 43 ORs, including several known pseudogenes, were different, such that mRNA expression declined in the mature OSNs relative to earlier stages. Protein and promoter sequence analysis of the atypical group did not uncover any obvious differences between them and more typical ORs. Nonetheless, the pattern of expression suggests that atypical ORs may be non-functional despite the lack of any obvious abnormality in the sequence analyses.

## Introduction

The gene family encoding olfactory receptors (ORs) is large, comprising on the order of a thousand or more loci in rodents and other macrosmatic mammals, and subject to complex regulation^1–3^. Expression of selected ORs, detected by labeling for a marker encoded by a bicistronic OR-IRES-marker construct or by anti-OR immunostaining, begins in immature olfactory sensory neurons (OSNs) a day or more after terminal mitosis^4–6^. As soon as they are detected in the adult olfactory epithelium (OE), the expression of any single OR is constrained to a swathe of the epithelium that occupies a fraction of the transverse axis of the OE, but generally extends along its full anteroposterior extent^7–10^. Furthermore, it appears that only one allele of one OR gene among the subset of OR loci available to a particular OSN on the basis of location across the epithelial map ends up as dominant once OSNs fully mature^11–14^. However, transcriptomic data suggest that more than one OR is expressed at detectable levels earlier in neuronal maturation^15,16^. Epigenetic suppression of the majority of loci combined with the release from suppression of the expressed OR(s) seems to be central to the process by which the single dominant allele emerges^17^.

In addition to the spatial and epigenetic regulation of the pattern of singular, monogenic, and monoallelic expression, the functional status of ORs also controls their stabilization as they reach full maturation. For example, a substantial proportion of the OR gene family, the size of which varies from species to species, can be classified as pseudogenes on the basis of sequence analysis and implied translation, as the proteins either terminate prematurely or exhibit frame shifts that preclude the normal 7-transmembrane domain structure^3,18^. Primary expression of an OR pseudogene is apparently not tolerated by OSNs, as they either switch from a non-functional OR to another one during the course of their differentiation or may die as shown by the lack of high level pseudogene expression in mature OSNs^19^. The existence of OR-switching from one OR gene to another, or an OR pseudogene to a non-pseudogene, has been demonstrated by elegant genetic experiments in mouse^20^. A negative feedback mechanism contributes to the establishment of singular expression and may be responsible for the switching that is observed^20–22^. During the profiling of the transcriptomes of marker-identified cell types in the mouse OE^23^, we found that the vast majority of expressed ORs are characterized by an increased level of expression as the sensory neurons differentiate and mature fully, which matches the results of previous gene expression profiling analyses^24–26^. However, we did observe a smaller set of ORs that exhibited an unexpected, atypical pattern of expression during the course of neuronal differentiation. This set of ORs reached maximal expression in the population of eGFP-labeled GBCs and immature neurons isolated from *Neurog1-eGFP* BAC transgenic mice. Expression levels declined within the population of eGFP-labeled mature OSNs isolated from heterozygous *ΔOMP-eGFP* knock-in transgenic mice. The behavior of these atypical ORs mimicked that of known pseudogenes but had not previously been classified as such and had no obvious truncations or frame-shift mutations. We characterize this set of atypical ORs here with respect to expression pattern, labeling by *in situ* hybridization, and analysis of gene and protein sequences by comparison with ORs whose expression are “typical” and matches expectations derived from the earlier work.

## Materials and Methods

### Animals

Wild-type F1 males were bred in house from parental strains (129S1/SvImJ × C57BL/6 J) acquired from The Jackson Laboratory. *Neurog1-eGFP* mice were generously provided by the GENSAT project^27^ and maintained as heterozygotes by successive matings to FVB/NJ mice or 129S1/SvImJ (The Jackson Laboratory). *ΔOMP-eGFP* mice were generously provided by Dr. Peter Mombaerts^28^ and maintained as homozygotes. Heterozygous *ΔOMP-eGFP* animals generated by outcrosses to CD-1 females were used. Heterozygous *ΔSox2-eGFP* mice on a C57Bl/6 J background were generously provided by Drs. Mahendra Rao and Larissa Pevny^29^ and were maintained as an inbred colony. *P2-ITL* mice were generously provided by Dr. Peter Mombaerts on a mixed 129 × C57BL/6 background^28^. All animals were housed in a heat- and humidity-controlled, AALAC-accredited vivarium operating under a 12:12-hour light-dark cycle. All protocols for the use of vertebrate animals were approved by the Committee for the Humane Use of Animals at Tufts University School of Medicine, where the animals were housed and experiments were conducted. All methods were performed in accordance with local guidelines and regulations. All mice were maintained on a 12-hour light/dark cycle with ad libitum access to food and water.

### Olfactory bulbectomy

The right olfactory bulb was removed by a technique previously described^30^. Mice were anesthetized by intraperitoneal injection of 0.6 mL/kg of an induction cocktail (43 mg/mL ketamine, 9 mg/mL xylazine, 1.5 mg/mL acepromazine), and followed as needed by 0.5 mL/kg of a maintenance dose (95 mg/mL ketamine, 1.9 mg/mL acepromazine). The bulb was exposed by removal of the overlying bone, the dura was lanced with a sterile 27- gauge needle, and the bulb was removed using a syringe attached to an aspiration pump. The ablation cavity was filled with Oxycel, and the animals were euthanized 3 weeks after the surgery.

### Cell dissociation, fluorescence activated cell Sorting (FACS), and sample preparation

Detailed FACS protocols have been reported from our lab and the details of cell types and their isolation by FACS are found in a previous publication^23^. Briefly, mice were deeply anesthetized by injection of a lethal dose of the induction cocktail described above and then perfused by intracardiac flush with low-Ca2+ Ringer solution (140 mM NaCl, 5 mM KCl, 10 mM HEPES, 1 mM EDTA, 10 mM glucose and 1 mM sodium pyruvate, pH 7.2). The olfactory epithelium (OE) was dissected into the septum and individual turbinate scrolls, and then incubated with 0.05% trypsin/EDTA (Gibco BRL) in low-Ca2+ Ringer solution for 15 min at 37 °C, followed by dissociation enzyme cocktail (collagenase/hyaluronidase/trypsin inhibitor/papain; 1 mg/ml, 1.5 mg/ml, 0.1 mg/ml, 15 µL/mL, respectively; Worthington Biochemical, Freehold, NJ and Sigma) in Ringer’s solution (140 mM NaCl, 5 mM KCl,10 mM HEPES, 1 mM CaCl2, 1 mM MgCl2, 10 mM glucose and 1 mM sodium pyruvate, pH 7.2) for 30 min at 37 °C with occasional trituration. Dissociated cells were treated with DNase I (Worthington) and subsequently filtered through 120 µm and 35 µm nylon mesh before staining and FACS.

FACS was performed on a MoFlo cell sorter (Cytomation Inc., Fort Collins, CO) at room temperature running Summit software (Dako) as described^23^. Cells from eGFP-expressing transgenic mice were dissociated as described above and resuspended in a solution containing HBSS (Ca2+/Mg2+ free; Invitrogen; Aukland, NJ) with 25 mM HEPES and 1 mM EDTA. An Innova 90 argon plasma laser (Coherent, Inc., Santa Clara, CA) was used to excite the cells at 488 nm and the samples were gated in FL1 to include only the eGFP (+) cells. Highest-expressing eGFP (+) cells from the *ΔSox2-eGFP* mouse encompassed sustentacular cells. Lower -expressing eGFP (+) GBCs were isolated from these animals following mechanical dissociation, which minimized contamination by horizontal basal cells (HBCs). Finally, horizontal basal cells (HBCs) were isolated following enzymatic dissociation and immunostaining with anti-ICAM (CD54) antibody (R&D systems, AF583). They were stained in suspension for 30 minutes at 4 °C, washed by centrifugation, and incubated with the appropriate allophycocyanin (APC)-conjugated secondary antibody before undergoing FACS.

Additional gating on forward and side scatter and on exclusion of propidium iodide (PI) was used to limit the sort to live cells^31^. Cells were sorted using a 70 µm nozzle at 60 PSI sheath pressure and collected in HBSS/HEPES/EDTA solution on ice. After collection, cells were spun for 10 minutes at 200 × g to pellet the cells.

### RNA purification, quality control, labeling and bead array processing

Cell pellets were resuspended in Buffer ZR (Zymo Research Quick RNA Microprep Cat#R1050) and the manufacturer’s instructions were followed exactly. RNA was eluted with water and DNase treated for 30 minutes at 37 °C. RNA was then purified using the DNA-free RNA kit from Zymo Research (Cat # R1013). Samples were run on an Agilent 2100 Bioanalyzer to determine quality and select intact, non-degraded samples. Three biological replicates (2–7 animals per replicate) were sent to the Keck Microarray Facility (Yale University) for RNA amplification, cRNA production, labeling, and hybridization to Illumina MouseWG-6 v2.0 bead arrays. Illumina Genome Studio software was used to export both raw data and background corrected, quantile–normalized data.

### Bioinformatic processing and analysis

Quality control and processing of microarray data was performed using the BioConductor packages lumi and limma for R^32–35^. Fluorescence intensity values were exported to Microsoft Excel in which data were formatted for subsequent analysis with in R, using standard K-means clustering and the MADE4 package for heatmap plotting^36^. The limma package was used to generate pairwise comparisons of the biological comparisons of interest. Gene lists for each comparison were filtered based on an adjusted p-value < 0.05. When smaller gene lists were required for analysis or study of specific candidates, an additional criterion of a fold-change greater than 2 was used.

Protein sequences of all the ORs were batch downloaded from NCBI based on the list of known olfactory receptors found in the SenseLab ORDB^37^. Evolutionary trace analysis^38^ was performed using the Evolutionary Trace tool available at: mammoth.bcm.tmc.edu/ETserver.html. Transmembrane domains were predicted using the ExPASy tool for TMpred, and sequence alignment and consensus sequences were generated using EMBL-EBI’s Clustal Omega tool. All promoter regions were assembled using UCSC Genome Browser, GRCm38/mm10 genome build, and defined as the 4000 bp of sequence upstream of the transcriptional start site. Binding motifs for Family 1 analysis were done using MatInspector for the respective transcription factor families. The predicted binding motifs for Ebf1 (MA0154.1), Emx2 (PH0027.1), and LHX2 (PH0092.1) were found on JASPAR2020. Promoter analysis using the MEME Suite were performed with default settings of MEME or FIMO prior to piping discovered motifs to MAST for searching and sorting.

*Tabula Muris* count data are publicly available and were analyzed using standard packages in R. ORs that had a total count of 5 or greater across the entire organ dataset and found in at least 3 single cells were considered expressed, providing higher sensitivity at the cost of increased false positives.

For statistical analyses, all data were first analyzed for normality using either the Kolmogorov-Smirnov test (for ANOVA) or the Shapiro-Wilk(for t-Test). All sets failed the normality test, and thus statistical significance was assessed using the non-parametric Kruskal-Wallis One Way ANOVA on Ranks, or the Mann Whitney Rank Sum Test.

Figures were assembled using either Adobe Illustrator CS5 or Adobe Photoshop CS5.

### Tissue processing

Animals were deeply anesthetized using the induction cocktail described above, transcardially flushed with PBS, and then perfused with either ice cold 4% paraformaldehyde (Fisher Scientific, Suwanee, GA) in 0.05 M sodium phosphate buffer, pH 7.2, or Zamboni’s Fixative. The OE was dissected and the tissue blocks were post-fixed under vacuum for 2 hours at room temperature. Tissues were then rinsed with PBS, equilibrated with 30% sucrose in PBS at 4 °C, and then frozen in OCT compound in liquid nitrogen (Miles Inc., Elkhart, IN). The olfactory mucosa was sectioned on a cryostat (Leica) in the coronal plane, 8 µm sections were collected on to “Plus” slides (Fisher Scientific) and stored at −20 °C for future use.

### *In situ* hybridization

Tissue was fixed in 4% paraformaldehyde that was prepared using DEPC-H_2_O and cryoprotected in 30% sucrose in DEPC-PBS before freezing; 12 µm thick cryosections were collected as described above. Hybridization and detection were performed according to published protocol^4,9^. DIG-labeled cRNA probes were produced by *in vitro* transcription of gel purified RT-PCR bands. The primers used to generate RT-PCR products were designed to incorporate a T7 RNA polymerase docking site for the generation of anti-sense probes and a Sp6 RNA polymerase docking site for the generation of sense probes. The probe sequences are as follows:

olfr1188 (ATT TAG GTG ACA CTA TAG cct gag atg ccc aac agc) and (TAA TAC GAC TCA

CTA TAG Ggc acc att gca agc aac ca),

olfr116(ATT TAG GTG ACA CTA TAG GGG CTC AGC AGG GAA CTT) and (TAA TAC GAC TCA CTA TAG GGT GGG GCA GGC AAG TAG AG),

olfr1508(ATT TAG GTG ACA CTA TAG tca tcc ttg ggt ctg aaa gg) and (TAA TAC GAC TCA

CTA TAG Ggt cag tgc aag cca gtt tga),

olfr282(ATT TAG GTG ACA CTA TAG CTC CCA CCT CCA CAC ACC) and (TAA TAC GAC TCA CTA TAG GGC AGA AGG CGG CAT GAG AT).

Briefly, sections were dried quickly with a hair-drier, rehydrated in PBS, permeabilized with 1% Triton-X in PBS followed by Proteinase K (PK) digestion (0.05 mg/mL, Roche 03115887001) in 1X PK buffer for 4 minutes at room temperature. Slides were rinsed in PBS twice, rinsed once quickly in water, and air-dried for 15 minutes. Digoxigenin-labeled RNA probes were heated to 65 °C for 5 minutes in hybridization solution (Sigma H7782-6ML) and applied to sections for hybridization for 16 hours at 55 °C in a humid box containing 5X SSC/50% formamide. Slides were washed twice with 2x SSC at room temperature and twice with 0.1X SSC at 60 °C for 30 minutes per wash. Sections were equilibrated in Tris Wash buffer, blocked for 30 minutes a 37 °C (1% blocking reagent in maleic acid buffer, Roche 11096176001). The sections were incubated for two hours at room temperature with alkaline phosphatase-conjugated sheep anti-digoxigenin (anti-DIG-AP) (Sigma-Aldrich). Sections were washed three times with Tris Wash buffer for 10 minutes each and equilibrated in AP buffer prior to chromogen reaction with 0.45% NBT/ 0.35% BCIP in AP buffer overnight at room temperature. Slides were then washed once in AP buffer and three times in H_2_O for 10 minutes each prior to de-colorization with 95% ethanol overnight.

For *in situ* hybridizations that were combined with immunohistochemistry, chromogen visualization of the anti-DIG-AP signal was followed by PBS washing. Sections were then blocked and stained according to immunohistochemical protocols described below.

### Immunohistochemistry

Standard laboratory protocols were used to detect expression pattern of individual protein in sections of the OE of the P2-ITL transgenic mice or of control mice subject to *in situ* hybridization^39^. Briefly, for the P2 mice, frozen sections were rinsed in PBS for 5 minutes to remove the OCT and then boiled in 0.01 M citric acid buffer (pH 6.0) for 10 minutes. After cooling, sections were rinsed with PBS briefly before incubating with block (10% serum/5% Non-fat dry milk/4% BSA/0.1% Triton X-100) for 15 minutes at room temperature. In all cases, the sections were incubated with primary antibodies overnight at 4 °C. Bound primary antibodies were visualized using fluorescently conjugated secondary antibodies from Jackson ImmunoResearch at 1:150 dilution following 1 hour incubation in block at room temperature. The following primary antibodies were used: 1:100 rat anti-NCAM (Abcam, ab19782 (H28-123))^23^, 1:500 rabbit anti-β-Gal (Cappel, #200–4136)^40^, and 1:150 chicken anti-eGFP (Abcam, ab13970)^41^.

## Results

### Hierarchical clustering of significantly regulated genes reveals distinct expression patterns

To examine the expression patterns of ORs as OSNs mature, we took advantage of eGFP-expressing transgenic mouse lines to FACS-isolate multiple distinct sets of cells from the whole of the olfactory epithelium: Sox2 (+) multipotent GBCs using a *ΔSox2-eGFP* mouse, Neurog1 (+) GBCs that function as immediate neuronal precursors as well as immature OSNs (due to eGFP perdurance) using a *Neurog1-eGFP* BAC transgenic line, and OMP (+) mature neurons using *ΔOMP-eGFP* reporter mouse^23^. We also isolated sustentacular cells and HBCs by FACS. Furthermore, we investigated the effect that an enhanced rate of neuronal production might have on OR choice and expression by isolating cells from the epithelium of Neurog1- and OMP-reporter strains 21 days after olfactory bulbectomy (OBX). The absence of the trophic support provided by the olfactory bulb abbreviates the lifespan of newly born OSNs, such that they die at or around the time they begin to express OMP. As a consequence, neurogenesis is accelerated relative to the uninjured OE^30^.

**Figure 1:**
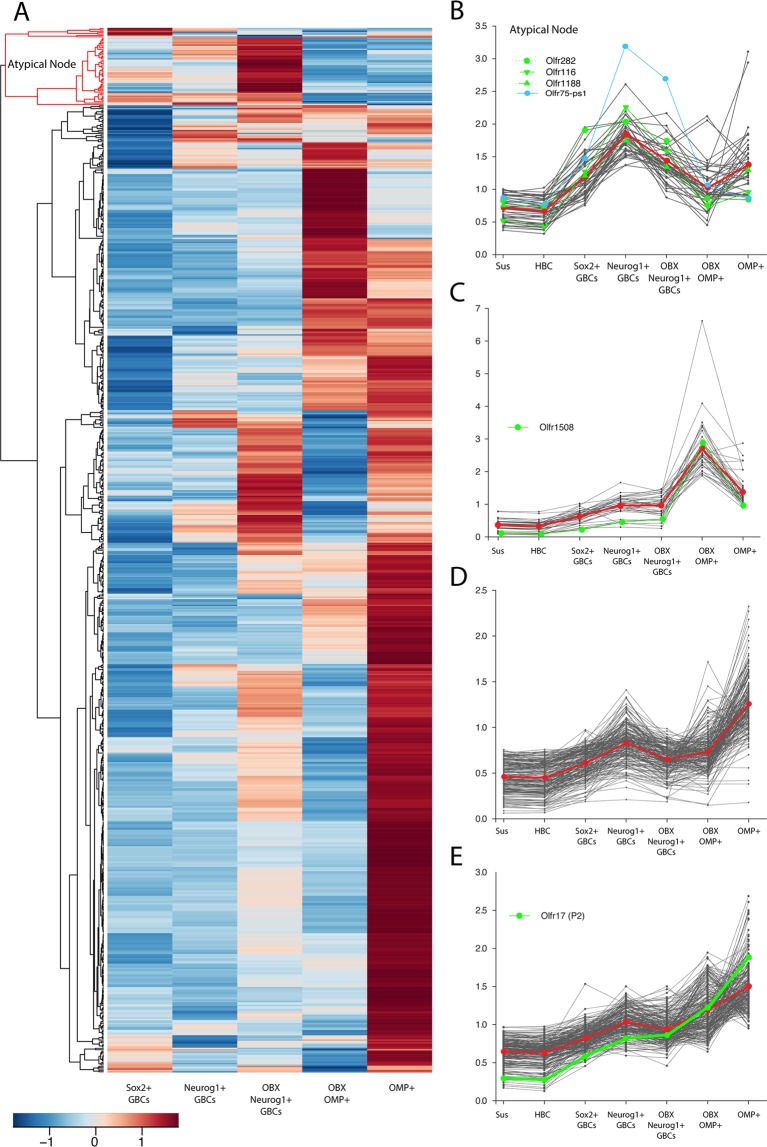
A subset of ORs genes are expressed in progenitor populations. All olfactory receptor (OR) genes were normalized to homogenized whole mucosa and hierarchically clustered based on their expression patterns in each cell type. (**A**) A row-normalized, hierarchically clustered heatmap depicting all ORs detected in the microarray. The node highlighted in red was explored in more detail due to their aberrant expression pattern. (**B–E**) In addition, K-means clustering was used to group ORs based on their expression patterns into 5 groups, of which 4 are depicted. In the graphs, each black line illustrates an individual OR probe. The red line delimits the centroid expression pattern of the group, while green and blue lines correspond to identified ORs selected for further analysis. Sox2+ = GFP-labeled cells in *ΔSox2-eGFP* mice; Neurog1+ = GFP-labeled cells in *Neurog1-eGFP mice*; OMP+ = GFP-labeled cells in *ΔOMP-eGFP* mice; OBX = olfactory bulbectomy. (**B**) The ORs encompassed by the red node at the top of the hierarchically clustered heat map (**A**) tend to have the highest level of expression in the GBC- and GBC plus immature neuron-enriched populations with and without bulbectomy (Sox2+, Neurog1+, OBX-Neurog1+). (**C**) The ORs in this group tend to have the highest level of expression in the OMP-OBX and OMP populations. (**D**) The ORs in this group tend to have a small peak in the Neurog1 population before reaching a maximum level in the OMP population. (**E**) The ORs in this group tend to have a steadily increasing level of expression during the progression, arriving at a maximum in the OMP population.

We constructed an average-linkage clustered, row-normalized heat-map of the expression of 1094 unique OR genes in the Illumina probe-set across the neurogenic progression (Fig. 1A). Inspection of the heat-map demonstrates that most of the ORs reach a maximum in one of the OMP (+) populations (either from control or post-bulbectomy epithelium), which is the expected or typical pattern of expression. However, a distinct set of OR genes deviated from that usual pattern, in that they were more highly expressed amongst the cells isolated on the basis of GFP-labeling from the *Neurog1-eGFP* mouse strain than in the *ΔOMP*-expressing cells (highlighted in red at the top of the heat map in Fig. 1A). As an alternative means of analysis, K-means clustering of the expression level of the individual probes was carried out relative to whole mucosa across the various cell types and established five distinct nodes, four of which are illustrated (Fig. 1B–E). Four of the five nodes, encompassing all but 43 of the OR genes, demonstrated an increase in expression level during the progression from the nadir established in sustentacular cells and HBCs, to *Δ*Sox2-eGFP (+) GBCs, to Neurog1-eGFP (+) GBCs and immature neurons from the epithelium of normal control (Neurog1Ctrl) and of bulbectomized (Neurog1OBX) mice, respectively, to *Δ*OMP-eGFP-expressing OSNs. For three of the four nodes manifesting peak expression in an OMP (+) population, the increase from the sustentacular cell baseline was substantial, and ranged from 2.6-fold to 7.1-fold (Fig. 1C–E). The fourth consisted of a large number of ORs (699/1094) that demonstrated only a slight rise in average expression (1.15-fold on average relative to sustentacular cells) along the neurogenic progression, suggesting that the numbers of neurons and/or the level at which the ORs in this node are expressed is low (not illustrated). However, one of the five nodes (highlighted in red at the top of the heat-map) encompassed a cohort of OR genes that peaked not in the OMP dataset but in a population upstream of the mature neurons – either in Sox2 (+) GBCs, or Neurog1-eGFP (+) cells, whether isolated from uninjured control OE or from the epithelium of bulbectomized mice (43/1094, or 4%) (Fig. 1B). We classify the ORs in this node as atypical, as the pattern of their expression was clearly distinct from the rest of the family. Using published RNAseq data from the Logan lab of whole mucosa vs. OMP-eGFP FACS enriched mature neurons, we confirmed that the expression levels of the atypical ORs were significantly reduced in OMP (+), mature neurons relative to the levels in whole mucosa by comparison with the ORs that exemplify the expected pattern of expression – average ratio of OMP(+)/whole mucosa for expression of typical ORs equals 1.8, while average ratio for atypical ORs is 1.05 (Yates corrected test of proportions, p < 0.001)^19^.

### Validation of olfactory receptor expression *in vivo*

**Figure 2:**
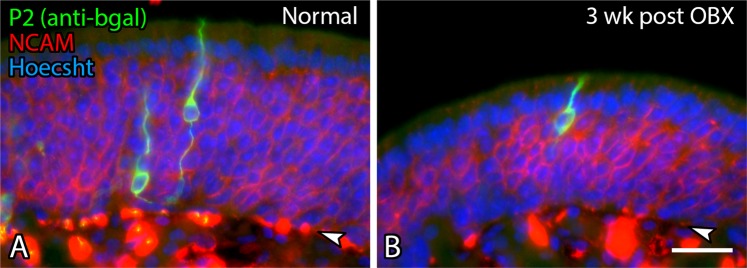
The expression of the P2 odorant receptor after bulbectomy. Sections of *P2-ITL* transgenic mice 3 weeks after unilateral bulbectomy (OBX). Frozen sections were stained for β-galactosidase (LacZ) to indicate P2 (*Olfr17)* neurons, anti-NCAM to mark all neurons, with the highest level of expression found in immature OSNs, and counterstained with Hoechst 33528 to label nuclei. Expression of the transgene on contralateral, unoperated, control side (**A**) and operated side 3 weeks after ablation (3 wk post-OBX) (**B**). The pattern shows β-gal (+) neurons in the apical portion of the OE after bulbectomy, paralleling the onset of *Olfr17* expression derived from the microarray data. Arrowheads indicate basal lamina. Scale bar in B is 25 µm and applies to both panels.

We next localized the expression of atypical and typical ORs within the epithelium itself. *Olfr17*, also known as P2, was chosen for analysis because it sits in one of the nodes composed of typical ORs (Fig. 1E), and has been studied extensively in multiple settings. The expression of this gene was examined on the unoperated and operated sides of P2-ITL transgenic animals three weeks after bulbectomy (Fig. 2). As reported previously^4^, P2 neurons, identified by β-gal staining, are found throughout the layer of mature neurons, with the rare basally-located immature β-gal (+) neuron. On the bulbectomized side, P2 neurons are concentrated near the apical aspect of the expanded lamina formed by the enlarged population of immature OSNs, which are more heavily NCAM (+) than mature OSNs, (Fig. 2), where differentiating OSNs are transitioning to short-lived OMP (+) mature neurons^4,30^. The localization of P2-ITL relative to the population of OSNs as a whole validates the microarray data, which shows an increase in *Olfr17* (P2) expression in the population of OMP-eGFP cells in the bulbectomized animals (OBX-OMP (+)) and a maximum level of expression in the OMP-GFP population in the control animals (OMP (+)) (Fig. 1E)^19^.

**Figure 3:**
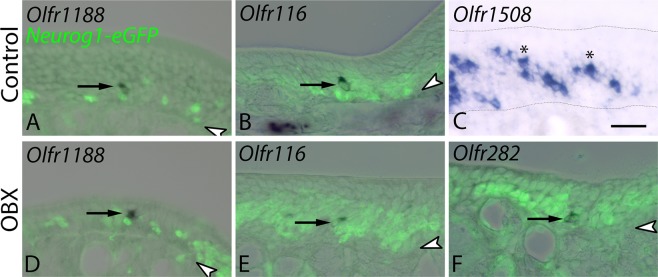
*In situ* hybridization of atypically regulated ORs. Sections from control mice and Neurog1–eGFP mice 3 weeks after unilateral bulbectomy were probed for expression of a subset of OR genes with peak expression in distinct populations as indicated by microarray data and highlighted in Fig. 1A,B. Sections from Neurog1–eGFP mice were co–stained with anti–GFP antibody. (**A–C**) Expression pattern of *Olfr1188*, *116*, and *1508*, respectively, on the unoperated side detected by *in situ* hybridization (ISH) followed by visualization with alkaline phosphatase conversion of NBT/BCIP (purple precipitate). Arrows in A and B indicate rare, basally situated neurons positive for Olfr*1188* and Olfr*116*, respectively. Asterisks in C mark some of the numerous *Olfr1508*(+) cells found throughout the apical to basal extent of the neuronal layer in control epithelium. (**D–F**) Expression pattern of *Olfr1188*, *116*, and *282* on the OBX side. Arrows highlight the rare, predominately basally located ISH-marked cells. In all panels, arrowheads indicate basal lamina. Scale bar in C is 25 µm and applies to all panels. Thin dotted line in C outlines the apical and basal borders of the epithelium.

In contrast, the atypical OR genes that were analyzed by *in situ* hybridization show a variable pattern of expression with respect to the number of cells labeled and position along the apical-to-basal axis of the epithelium. By microarray, *Olfr1188, Olfr116*, and *Olfr282* were all expressed at the highest level in the population of Neurog1-eGFP (+) population in control animals (Neurog1 (+)) (Fig. 1B). In all three cases, cell bodies labeled by *in situ* hybridization were infrequent, primarily located in the basal half of the epithelium, and confined to the population of Neurog1-eGFP (+) GBCs and immature OSNs in both the uninjured and bulbectomized mice (Fig. 3). *Olfr1508* (*MOR244-2*) is included as an additional example of a highly-expressed, typically-regulated OR, since it is more highly expressed in OMP (+) OSNs than in the Neurog1-eGFP populations (Fig. 1C), which pattern is similar to that of P2. In contrast to the atypical ORs, the neurons labeled by *in situ* hybridization for *Olfr1508* are distributed through the depth of the uninjured OE; since immature OSNs in the uninjured OE are limited to a single layer just superficial to the basal layer of the epithelium, the vast majority of the *Olfr1508*-labeled OSNs are mature (Fig. 3), which contrasts markedly with the atypical ORs.

### Sequence analysis of atypical ORs

While the regulation of the atypical OR genes does not fit with the pattern expected for functional ORs, it does match the pattern expected for ORs that are classified as pseudogenes on the basis of their DNA sequence, which corresponds to roughly 20% of the OR gene family in mice^2^. The expression of pseudogenes appears to be subject to gene switching, such that the expression of a pseudogene can be “switched-off/out” and that a different, functional OR can be “switched on/in”;^20–22^ the switch seems to take place as the OSNs are maturing. Thus, as neurogenesis and neuronal differentiation progress, the expression pattern for a pseudogene is predicted to closely resemble that of the ORs that fall into the “atypically-regulated” node of the heat-map presented here (Fig. 1A,B). Indeed, amongst the atypical ORs, 2 of the 43 have been previously classified as pseudogenes, i.e., non-functional ORs (Ensembl assembly GRCm38.p5) (for example *Olfr75-ps1*, Fig. 1B)^2,3,42^. The other atypical ORs in the oddly-regulated node have not been previously designated as pseudogenes. These 41 genes encompass 31 OR receptor gene subfamilies^2,3^.

**Figure 4:**
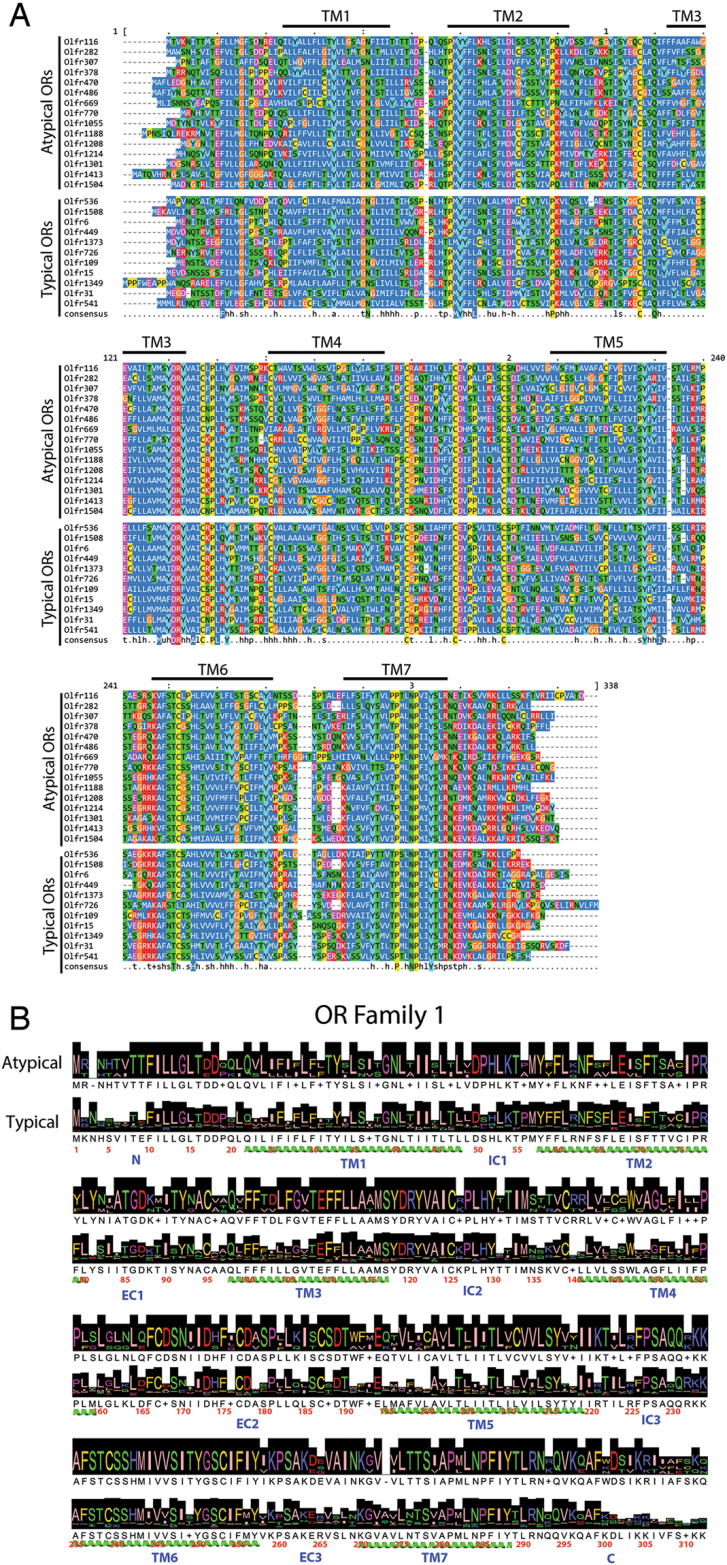
Alignment of ORs in the atypical and highly typically regulated node. (**A**) The ClustalOmega protein sequence alignment tool^52^ was used to align the protein sequences for all members of the atypically regulated node vs. typically regulated node from Fig. 1B,C, respectively, that satisfied the expression level criterion described in the text. The alignment is depicted using MView^53^ and colored using the NARDI color palette. Transmembrane domains (TM) domains are indicated by black bars and labels above the alignment. (**B**) A further refinement of the alignment analysis comparing atypical vs. typical ORs in Family 1^2^. Height of the black shading represents percent identity, while transmembrane regions are represented by helices below the sequence.

Since the majority of these atypical OR genes were not previously recognized as pseudogenes, we conducted an *in silico* analysis to see if there might be an overt sequence variation that could predict differential regulation of their expression. To increase the sensitivity, we constrained our analysis in two directions. First, we focused on ORs that were characterized by a log2 fold-change of 1.5 or greater (2.83 fold) relative to the HBC and sustentacular cell expression nadir to minimize the signal to noise problem inherent in a bulk analysis of this sort. We compared those in the atypical group that satisfied the fold-change criterion (Fig. 1B) with ORs in the closest typical node that also meet the expression level criterion (Fig. 4A). Second, we focused on ORs in OR Family 1^2^, which contained the largest number of atypical ORs (4 out of 29 total ORs in family 1), and performed sequence conservation and consensus analysis, including prediction of the transmembrane helices using TMpred (Fig. 4B). Neither overt mutation – e.g., an early truncation – nor gross distortion of transmembrane helices was observed.

To ascertain whether the protein sequences of the atypical ORs differ in some subtle manner from typical ORs, we performed a detailed evolutionary trace analysis of all of the murine ORs. We noted that the very first division separated 107 ORs from the rest and that this group contained only 1 oddly-regulated OR out of 20 total identified (data not shown). No significant mutation pattern was evident to differentiate that OR from the rest. Furthermore, the larger group of atypical ORs does not segregate from the rest on protein sequence grounds.

**Figure 5:**
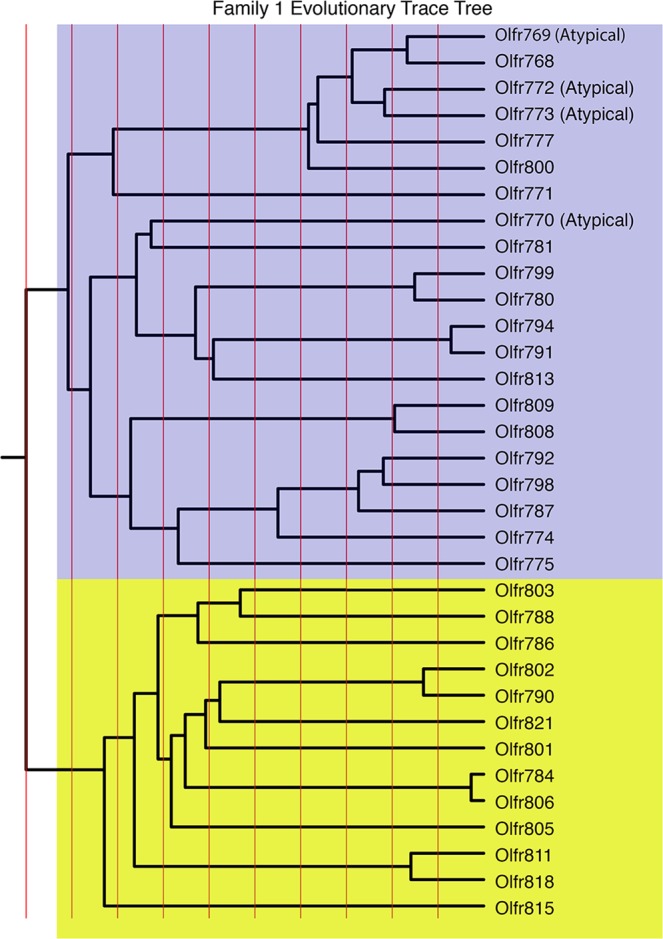
Evolutionary trace of Family 1 olfactory receptors. The murine Family 1 olfactory receptors contained 29 typically regulated receptors, and 4 atypical ones. The first phylogenetic division split the family into two groups, containing 20 and 13 receptors each (lavender and yellow background shading, respectively). The group containing 20 receptors contains all four atypical receptors.

We then subjected Family 1 to an evolutionary trace analysis on its own. As in the full OR analysis, the very first partition divided family 1 ORs into two groups. In contrast to the overall analysis, all four of the atypical ORs, out of a total of 20 ORs in that group, were confined to one of the partitions (Fig. 5, lavender box). A detailed conservation analysis of family 1 showed that within residues 39–71 (part of transmembrane domain 1), the GNLTII_LT motif is highly conserved amongst the typical ORs (where “_” corresponds to T45, S45, or I45). However, the sequence of three of the four atypical ORs – GNL**A**IISL**I** (2 ORs) and GNLTII**L**LT (1 OR)– diverged in that motif (divergent residues are bolded). No other mutation pattern was discernible above background.

To narrow further the focus, we also compared OR pairs that included one atypical OR and a closely related OR whose expression fit the expected pattern; the members of the selected pairs grouped together at partition 8 or after in the full OR evolutionary trace analysis. We identified 9 such pairs, which were subjected to pairwise alignment. Sites that differed across at least 4 pairs were compared further (Table 1). Of note, TM1 again contained mutations (positions 24, 27, and 31) that differ between members of the pairs and could present on a single face of the predicted alpha helix, each a single turn of the helix above the next. It is important to note that all atypical ORs from Family 1 were excluded from this pairwise analysis because there was no suitable normal OR partner that satisfied the Partition 8 criterion. The mutations noted in the preceding paragraph at residues 42 and 47 in the atypical ORs of family 1 are unlikely to be on the same face of the helix as the others. As this type of analysis will enrich for regions of high variability, we cannot distinguish whether this location is simply a permissive variable region, or whether it plays a special role (as yet undefined) in the oddly-regulated, presumably nonfunctional status of an OR.

**Table 1:** Result of pairwise comparison of atypical ORs with closely tied typical ORs residing in the same clade after partition 8 of the evolutionary trace analysis of murine ORs (Fig. 5).

Residue	Site	# swaps	Mutations
24	TM1	4	C→G, V→I (2), L→I
27	TM1	5	S→C, S→I, V→F, L→F, V→L
31	TM1	4	V→T, A→T, V→S, V→L
86	EC1	4	R→G, Q→K, K→E, T→S
104	TM3	4	W→A, I→F, F→Y, L→F
132	IC2	4	I→V, T→S (2), S→F
145	TM4	4	A→S, L→I, V→I, I→V
210	TM5	4	L→I, A→L, I→F, F→S
226	IC3	4	M→I (2), V→F, T→A
308	C-term	4	R→L, I→L, V→M, A→S

Each listed residue contained a mutation in at least 4 atypical-typical OR pairs that satisfy the post-partition 8 criterion. Residue numbering is based on the total consensus sequence. Numbers in parentheses mark the number of times any particular swap occurred.

### Promoter sequence analysis of oddly-regulated ORs

Because there appeared to be no striking differences at the amino acid level, we next investigated the upstream promoter sequences of Family 1 ORs. It is important to acknowledge the caveat that long-range modulators would likely escape detection under this analysis, including the well-studied H-region^21^. First, we analyzed whether the previously identified canonical OR regulatory binding motif families: HBOX (which includes Emx2), LHXF (which includes Lhx2), and NOLF (which corresponds to the Olf1/Ebf1 family) were differentially found in these sequences^43–46^.

**Figure 6:**
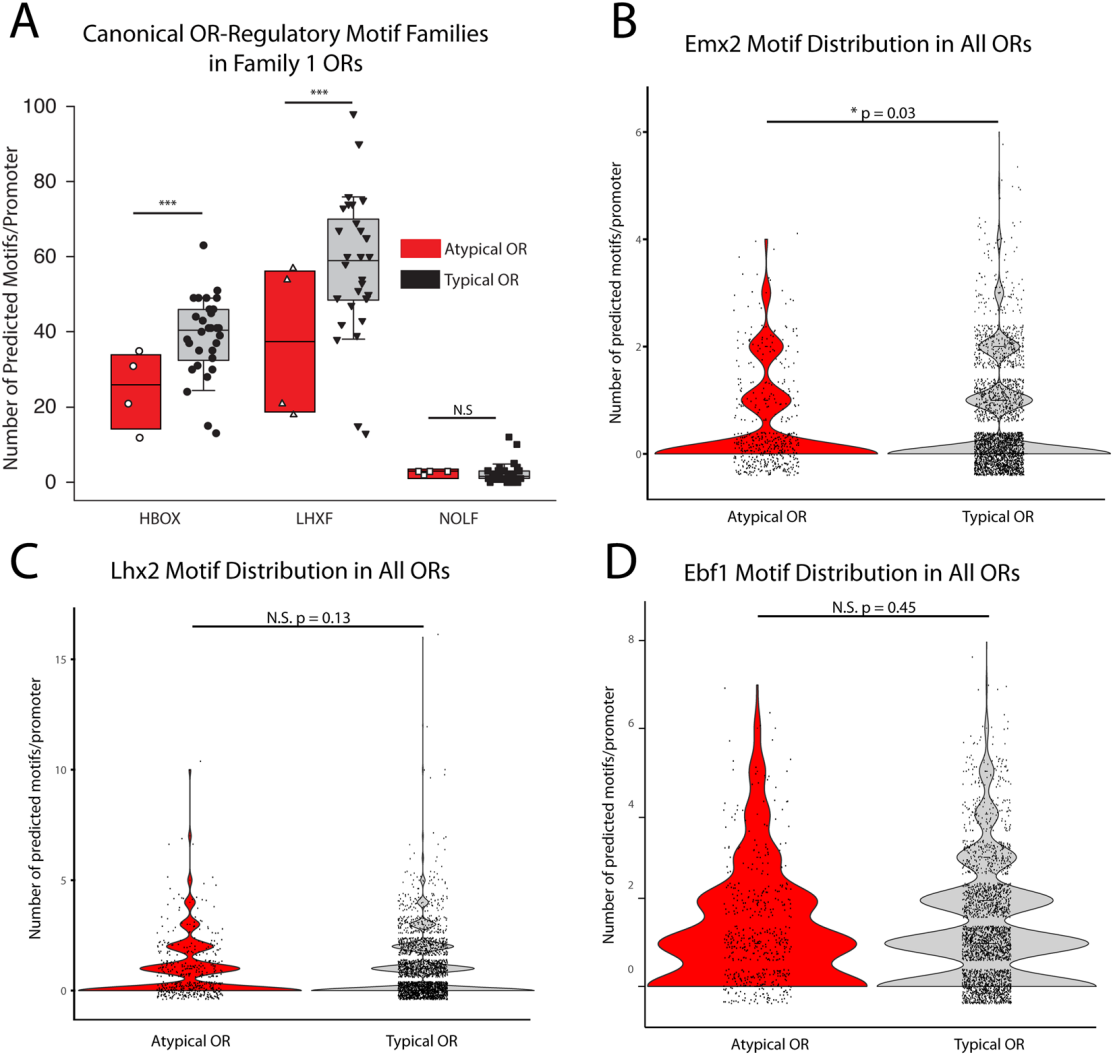
Enrichment of canonical OR-regulatory motifs in the upstream promoter regions of atypically vs. typically expressed ORs. (**A**) Genomatix was used to identify motifs that are known to participate in the regulation of OR expression, for example HBOX (which includes Emx2), LHXF (which includes Lhx2) and NOLF (the Olf1/Ebf1 family)^44^. The first two demonstrate significant differences in number of identifiable motifs between atypically expressed and typically expressed ORs of Family 1. (**B–D**) Violin plots of the number of predicted motif sites using FIMO for (B) Emx2, (C) Lhx2, and (D) Ebf1 found in all atypical ORs vs typical ORs. Each individual dot represents a X and Y jittered point to allow visualization of density of points.

Using MatInspector analysis and the Genomatix motif library for these three families, we analyzed all ORs found in Family 1 and found that there was indeed a significant reduction in HBOX and LHXF but not NOLF motif family sites in atypical ORs compared to typical ones (Fig. 6A). To extend this analysis to encompass all ORs in the murine genome, we restricted further analysis to the specific motif binding sites for Ebf1 (NOLF), Emx2 (HBOX), and Lhx2 (LHXF), as they are known to be the specific transcription factors within their family that regulate OR regulation. We again saw a significant decrease in Emx2 (HBOX) predicted motif binding sites (Fig. 6B), though Lhx2 (LHXF) motif binding sites only trended towards significance (Fig. 6C). Similar to the previous analysis, there was no difference in Ebf1 (NOLF) motif binding sites (Fig. 6D)

**Figure 7:**
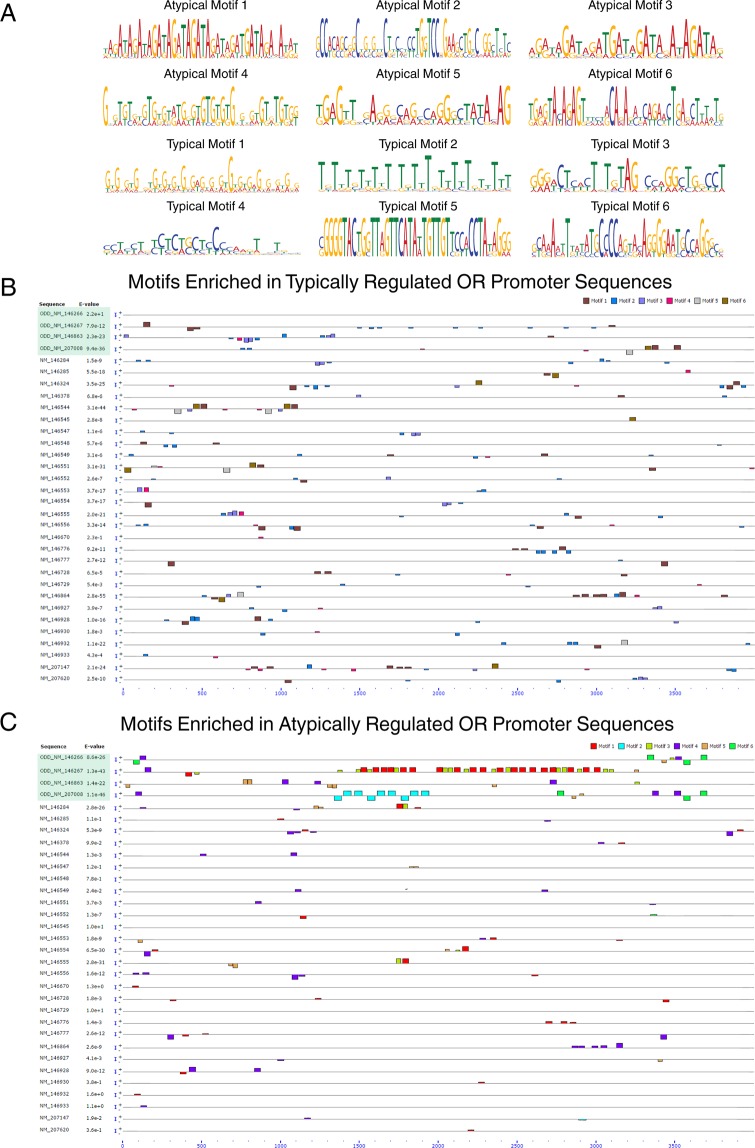
Identification and mapping of *de novo* identified motifs in promoter regions. (**A**) Nucleotide sequences of the most common MEME-identified motifs in the promoter regions of atypically and typically regulated ORs (6 each). Note the absence of sequence overlap between motifs classified as atypical vs. typical. (**B**) Motifs enriched in typically regulated ORs are mapped across 4 kb of the promoter regions upstream of the transcription start site for each OR in Family 1; each “typical” motif was localized using MAST. Bar height represents confidence in the sequence matching predicted motif consensus sequences. Bars above the line represent motif sites on the positive strand, while bars below the line represent motif sites on the negative strand. (**C**) A similar mapping was performed with the motifs identified in atypically regulated OR promoters and depicted in the same way as (B).

**Figure 8:**
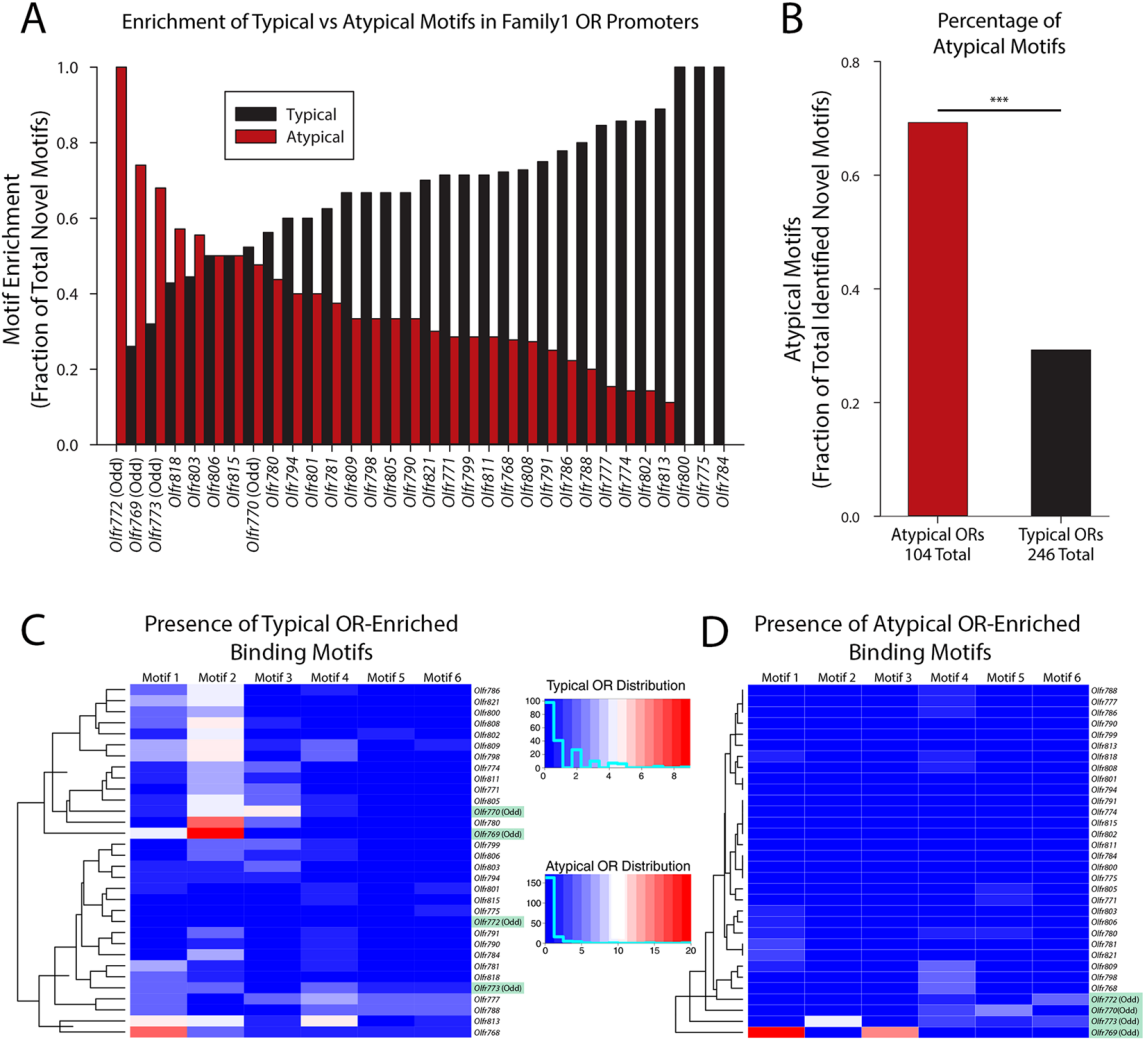
Distribution analysis of *de novo* identified motifs. (**A**) Enrichment of typical vs atypical motifs plotted as a percentage of total motifs found in each promoter region showing significant skewing such that atypical motifs are more frequent in atypically regulated ORs (Odd). (**B**) Summarized percentages of overall occurrences of atypical motifs in either group shows significant preference for atypical motif being found in atypically regulated ORs. (**C**) Hierarchically clustered heatmap depicting the frequency distribution of each typical motif in each OR in family 1. Atypically regulated receptors are marked in green and show a scattered distribution. (**D**) A similar heatmap to (C) but plotting atypical motifs, showing that atypical receptors clustered with high incidence of atypical motifs and a bimodal distribution of atypical motifs.

To prevent bias towards previously studied transcription factor binding sites, we also used MEME (Multiple Em for Motif Elicitation) to discover novel motifs present in Family 1 OR promoters of atypical vs. typical ORs in the 4 kb of sequence upstream of their transcription start site^47^ (Fig. 7A). The top 6 motifs in each group were mapped across all sequences using MAST (Motif Alignment & Search Tool); the atypical ORs in family 1 are highlighted in green (Fig. 7B,C)^47^. The top 6 motifs found in the promoters of the typically regulated ORs of Family 1 did not show any sequence overlap with the top 6 motifs in the atypical ORs; indeed, the sequences of all 12 motifs were highly divergent. Next, we assessed the enrichment for each of the 12 motifs in every Family 1 OR individually. As expected, the distribution was skewed across the set of Family 1 ORs, such that motifs identified upstream of atypical ORs (Atypical-upstream motifs) were found predominantly in atypical OR genes, and, vice-versa, for motifs upstream of typical ORs (Typical-upstream motifs) (Fig. 8A). That association of atypical motifs with atypical ORs and vice-versa was evident when graphed as a total percentage across all Family 1 ORs (Fig. 8B). The frequencies of the various motifs across the Family1 promoters were determined separately for typical and atypical motifs. Clustering according to the number of typical motifs in the upstream region did not group typical ORs closely; instead, they were intermingled with atypical ORs (Fig. 8C). Correspondingly, the number of OR motifs characteristic of typically-regulated ORs were broadly distributed (Fig. 8C). On the other hand, clustering according to the number of atypical motifs ended up grouping the atypical ORs, which stood out by virtue of the large number of atypical motifs present in the upstream region of several of the atypical ORs themselves (Fig. 8D).

### Atypical ORs are not preferentially expressed in non-olfactory tissue

**Figure 9:**
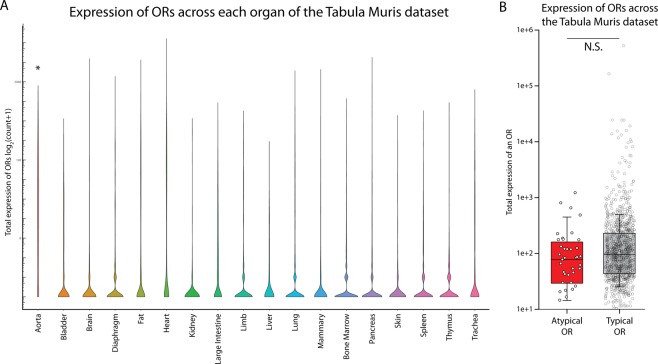
Expression of atypical ORs across the Tabula Muris dataset. (**A**) Violin plot of all olfactory receptors expressed in non-olfactory tissue as assayed by the Tabula Muris dataset. (**B**) Analysis of atypical vs typical OR expression across the Tabula Muris dataset reveals no differential expression for atypical ORs in non-olfactory tissue.

Olfactory receptors are expressed in many non-olfactory tissues, playing a diverse set of roles ranging from energy metabolism to regulation of migration^48^. Given the aberrant expression pattern of these atypical ORs, we investigated whether these atypical ORs were actually preferentially expressed in other tissues. None of the atypical ORs identified here have been directly investigated and, thus, no reported extra-olfactory role. Nonetheless, we data-mined the recently published *Tabula Muris* dataset, which covers 20 mouse organs at a single-cell transcriptomic level^49^. We were able to successfully data-mine 19 of the 20 organs (excluding tongue and combining all of the brain fractions into one) and examined the expression of all known ORs in each organ (Fig. 9A). The aorta (median count = 17) expressed a significantly higher number of olfactory receptors compared to the other organs (median count = 0) (Kruskal-Wallis One-Way-ANOVA, p < 0.001). However, within the aorta, there was no differential expression of atypical (median count = 24) vs typical OR (median count = 36) (Mann-Whitney Rank Sum p = 0.190). We extended this to cover all of the organs we could analyze in the *Tabula Muris*, and found no systematic preference for an atypical OR (median count = 78) over a typical OR (median count = 96) (Mann Whitney Rank Sum p = 0.071), though it may trend slightly, in fact, towards typical ORs (Fig. 9B).

## Discussion

The microarray analysis of gene expression during the neurogenic progression from multipotent Sox2 (+) GBCs to the OMP (+) mature OSNs demonstrated that expression of a subset of OR genes peaks in more upstream and undifferentiated cells types as compared to the vast majority of ORs that reach a maximum in OMP (+) populations, as expected from previous analyses^24,25^. The OR genes that are characterized by an atypical pattern of expression reach a maximum in advance of full neuronal maturation and OMP expression; these atypical ORs may represent a previously unidentified population of functionally inactive genes or pseudogenes that had not been previously detected by sequence analysis^2,3^. Indeed, we show that the pattern of expression of ORs classified as pseudogenes on the basis of truncation or frame-shift closely matches that of the atypical ORs reported here (e.g., *Olfr75-ps1*, Fig. 1B).

The number of expressed OR genes detected here – 1094 – compares favorably with previously published profiles of whole OE (ORs detected = 1087)^19^ as does the numbers identified in either Neurog1-eGFP (+) cells, which includes GBCs and immature OSNs due to eGFP perdurance, or mature, OMP (+) OSNs^24,25^. The expression patterns of some of the ORs that we analyzed here have also been reported previously in the published literature^24–26^. Of the 43 ORs that are expressed with an atypical pattern, 16 were detected in whole OE mRNA by hybridization to the Agilent Mouse Genome 4 × 44 K chip^26^; all of them were downregulated in the epithelium of AC3 knockout mice. It is worth noting that three *Olfr* pseudogenes (classified as such on the basis of their nucleic acid sequence) were also detected, and the expression of each was also reduced in the AC3 knockout-epithelium^25^. Of the remaining 1051 ORs that were not classified as being atypically expressed, 413 were detected in whole OE mRNA^25^. The majority (400/413) of them were also down-regulated as a consequence of AC3 knockout. Likewise, a handful of the typical ORs illustrated in Fig. 1 are listed among the ones assigned to mature and/or immature neurons previously using hybridization to the Affymetrix M430v2.0 GeneChip, including *Olfr15* and *Olfr1508*; all reach maximal levels in the mature OSNs^24,25^. In cases where an OR can be assessed in both immature and immature neurons, the published observations^19,24,25,46^ are consistent with our findings presented here.

The number of atypical ORs that are expressed in progenitors and/or immature OSNs but become less abundant as the neurons reach full maturity is not insignificant. That their expression is not maintained in mature OSNs suggests that the atypical OR genes may encode a protein that is incapable of the initiating the negative feedback signal that is characteristic of more typical OR proteins^21,22^. As a consequence the atypical OR may be “switched-out” and replaced by another, more typical OR in OSNs that persist and innervate glomeruli; that scenario has been observed when OR alleles are replaced by a non-OR^20^ or when pseudogenized^50^. In the latter case, the glomerular labeling is limited probably because expression of the tag from the switched-off locus decays^50^. Nonetheless, we cannot rule out the possibility that the neurons expressing an atypical OR die around the time that they begin to express OMP.

It is worth noting that OSN activity is not a prerequisite for prolonged survival. For multiple OR-defined OSN types, silencing by OCNC1 channel knockout does not prevent the neurons from establishing and maintaining a glomerular territory that is shared with OSNs with an intact channel^51^. The establishment and maintenance of mixed channel-knockout and channel-intact OSN-innervated glomeruli is increased in number with naris occlusion and the accompanying sensory deprivation. Therefore, activity dependence is a feature of the competition for intraglomerular, not interglomerular, territory^51^ and the accompanying putative trophic support^30^. Thus, the most established explanation for the lack of atypical OR expression is their replacement by a more typical OR during neuronal maturation.

Indeed, it is irrelevant to their designation as atypical ORs whether the neurons that initially express them switch ORs and survive to maturity or not. Rather, the identification of apparently intact OR alleles whose expression is not maintained may offer a novel tool of assessing how a typical OR is able to suppress other alleles or to provide insights into typical OR function, since the atypical ORs lack the gross sequence abnormalities like premature termination or frame-shift mutation characteristic of known pseudogenes. Clearly, analysis of the predicted protein sequence and of the region of DNA upstream of the transcription start site did not distinguish atypical from typical ORs in our dataset or suggest why the two sets might differ. On the other hand, analysis of the region of DNA upstream of the transcription start site found a slight but significant decrease in the number of HBOX or LHXF motifs—canonical OR-regulatory motifs. This is consistent with the importance of Emx2 and Lhx2 in OR gene choice. However, why a decrease in binding of these transcription factors would result in mis-expression rather than decreased expression has yet to be explained. We did also find a decrease in other, novel motifs that are generally found in typical ORs accompanied by a concomitant increase in novel motifs found primarily in atypical ORs. It may be that the combination of altered promoter structure prevents OR stabilization and compels switching out of the atypical receptors. Nonetheless, the current findings complement recent work indicating that pseudogenes are expressed in embryogenesis alongside canonical OR genes^5^. While functional studies are still lacking, the data from this study and the work published by the McClintock lab^24,25^ suggest that sequence data may not be sufficient to determine with certainty whether a given OR is functional or not.
